# The Targeting of Noncoding RNAs by Quercetin in Cancer Prevention and Therapy

**DOI:** 10.1155/2022/4330681

**Published:** 2022-05-24

**Authors:** Man Wang, Xinzhe Chen, Fei Yu, Lei Zhang, Yuan Zhang, Wenguang Chang

**Affiliations:** Institute for Translational Medicine, The Affiliated Hospital of Qingdao University, College of Medicine, Qingdao University, Qingdao 266021, China

## Abstract

The dietary flavonoid quercetin is ubiquitously distributed in fruits, vegetables, and medicinal herbs. Quercetin has been a focal point in recent years due to its versatile health-promoting benefits and high pharmacological values. It has well documented that quercetin exerts anticancer actions by inhibiting cell proliferation, inducing apoptosis, and retarding the invasion and metastasis of cancer cells. However, the exact mechanism of quercetin-mediated cancer chemoprevention is still not fully understood. With the advances in high-throughput sequencing technologies, the intricate oncogenic signaling networks have been gradually characterized. Increasing evidence on the close association between noncoding RNA (ncRNAs) and cancer etiopathogenesis emphasizes the potential of ncRNAs as promising molecular targets for cancer treatment. Available experimental studies indicate that quercetin can dominate multiple cancer-associated ncRNAs, hence repressing carcinogenesis and cancer development. Thus, modulation of ncRNAs serves as a key mechanism responsible for the anticancer effects of quercetin. In this review, we focus on the chemopreventive effects of quercetin on cancer pathogenesis by targeting cancer-relevant ncRNAs, supporting the viewpoint that quercetin holds promise as a drug candidate for cancer chemoprevention and chemotherapy. An in-depth comprehension of the interplay between quercetin and ncRNAs in the inhibition of cancer development and progression will raise the possibility of developing this bioactive compound as an anticancer agent that could be highly efficacious and safe in clinical practice.

## 1. Introduction

Quercetin (3,3',4',5,7-pentahydroxyflavone) is a polyphenol compound and the most ubiquitous flavonoid in fruits, vegetables, and medicinal plants [1]. They are found in a wide range of foods including apples, berries, capers, grapes, onions, shallots, tea, and tomatoes, as well as barks, flowers, leaves, nuts, and seeds [2]. Quercetin is one of the most prevalent flavonoids in the human diet. The biological effects of quercetin have been extensively studied. It has been generally accepted that quercetin exhibits antioxidant, anti-inflammatory, antimicrobial, and antiparasitic activities [3–6]. In recent years, quercetin has garnered attention for its cancer chemopreventive and chemotherapeutic properties. Quercetin plays a key role in affecting the hallmarks of cancer [7]. Specifically, quercetin facilitates cell cycle arrest and apoptosis in cancer cells. Quercetin regulates the proliferation, invasion, migration, and chemotherapeutic sensitivity of cancer cells. In addition, quercetin has an impact on the metabolism of chemotherapeutic agents. Although a number of literatures on the anticarcinogenic actions of quercetin have been published earlier, its underlying mechanisms are still not completely understood.

Noncoding RNAs (ncRNAs) have emerged as vital mediators in cancer chemoprevention by naturally occurring bioactive compounds since ncRNAs are able to modulate a variety of proteins and signaling pathways [8, 9]. Nowadays, ncRNAs have become one of the hottest topics in the field of biomedical science. ncRNAs form over 90% of the RNAs transcribed from the human genome [10]. With the rapid development of next-generation sequencing techniques, tens of thousands of ncRNA species have been discovered, but most of them are yet to be studied. Based on their size, ncRNAs can be subdivided into small ncRNAs and long ncRNAs (lncRNAs) [11]. Small ncRNAs are shorter than 200 nucleotides (nt) in length, and small interfering RNAs (siRNAs), microRNAs (miRNAs), and piwi-interacting RNAs (piRNAs) belong to this subfamily [12]. lncRNAs are a class of functional RNAs with length longer than 200 nt with no or limited protein-coding potential [13]. Particularly, circular RNAs (circRNAs) are a type of lncRNA molecules that are characterized by a covalently closed ring structure [14]. ncRNAs have been reported to be aberrantly expressed in cancer and act as critical participants in carcinogenesis and cancer progression [15, 16]. The disclosure of the roles of ncRNAs in cancer development has added new layers of complexity in comprehending the fine-tuned modulation of cellular processes contributing to cancer. It is thus proposed that ncRNAs represent promising therapeutic targets for the treatment of cancer. Several clinical studies have been commenced to explore the therapeutic efficacy of ncRNA-based anticancer agents [17–19]. Notably, accumulating evidence suggests that quercetin functions to alter the expression and function of ncRNAs associated with cancer pathogenesis [20, 21]. Quercetin can govern oncogenic or tumor-suppressive ncRNAs [22]. Moreover, quercetin exhibits the ability to orchestrate multiple cancer-relevant signaling pathways, such as the apoptotic pathway and the Wnt/*β*-catenin signaling cascade, via directly acting on different kinds of ncRNAs [23, 24]. The findings mentioned above substantiate the causal involvement of ncRNAs in the anticancer mechanisms of action of quercetin. Based upon the evidence gathered so far, this review summarizes the knowledge regarding the beneficial roles of quercetin against cancer mediated by ncRNAs. A better understanding of molecular mechanisms underpinning the protective role of quercetin in cancer is critically important before it can be recommended as an anticancer agent.

## 2. Dietary Source of Quercetin

Naturally occurring quercetin is widely distributed in plants, such as Amaryllidaceae, Brassicaceae, Capparaceae, Ericaceae, Liliaceae, and Rosaceae. Specifically, food-based sources of quercetin include fruits (e.g., apples, berries, cherries, and grapes), vegetables (e.g., asparagus, celery, onions, and peppers), and plant-derived beverage (e.g., red wine and tea) [25]. Quercetin mainly occurs in glycosidic forms such as hyperoside (quercetin-3-O-galactoside), isoquercitrin, and rutin, and it is also present as the free aglycone [26]. The constitutive pattern of quercetin glycosides differs among diverse foods. For instance, apples mainly contain quercetin-3-O-galactoside, quercetin-3-O-glucoside, quercetin-3-O-rhamnoside, and quercetin-3-O-rutinoside, while onions comprise quercetin-3,4'-diglucoside and quercetin-4'-glucoside [27]. In addition, medicinal plants are also rich sources of quercetin, such as *Ginkgo biloba*, *Hypericum perforatum*, and *Sambucus canadensis* [2].

## 3. Metabolism of Quercetin

The bioavailability and metabolism of quercetin have been well defined. Only a small quantity of quercetin is absorbed in the stomach, and the main site of absorption is the small intestine [28]. Quercetin predominantly occurs in glycosidic form after oral intake. Continuous consumption of quercetin-containing diet markedly elevates the concentration of quercetin in plasma. Quercetin is primarily present as its glycosidic form in human blood. Notably, the absorbed unit of quercetin is the aglycone itself. Therefore, the glucosides attached to quercetin are cleaved before absorption into the enterocytes. The type of glycoside connected with quercetin has an effect on its bioavailability. Rutin and other quercetin glycosides that are connected with oligosaccharide or polysaccharide are resistant to hydrolysis by intestinal hydrolases, and thus, they enter the large intestine unchanged where they are hydrolyzed by gut microbial hydrolases [28]. On the other hand, quercetin monoglycosides such as isoquercitrin (quercetin 3-glucoside) and quercetin-4'-glucoside are absorbed in the upper part of the intestine (the small intestine) after cleavage of the glycosidic bond by intestinal glycosidases, leading to the liberation of deconjugated aglycone [29]. The released lipophilic aglycone is mainly absorbed by intestinal epithelial cells by passive transport. The quercetin metabolites are transported by the hepatic portal vein to the liver, where they are further metabolized, including O-methylation, glucuronidation, and sulfation [30]. The metabolism of quercetin leads to the formation of phenolic acids in the small intestine and colon, along with the breakdown of quercetin skeletal structures. Quercetin and its metabolites are mainly excreted in the intestine, and only a small portion is excreted in the urine by the kidneys [31]. Overall, quercetin becomes metabolized in multiple organs including the small intestine, colon, liver, and kidney following absorption.

## 4. The Biological Function of Quercetin

Existing evidence has proven that the antioxidant activity of quercetin is predominantly manifested through its impact on glutathione (GSH), antioxidant enzymatic activity, reactive oxygen species (ROS), and various signaling cascades (e.g., NF-*κ*B, AMPK, and PI3K/Akt) [32]. GSH can defend against oxidative stress by acting as a hydrogen donor in the neutralization of hydrogen peroxide [33]. In vivo experimental results showed that high intake of quercetin significantly enhanced GSH production in the liver [34]. Quercetin also stimulated the expression of the antioxidant enzymes glutathione peroxidase 1 (GPX1), catalase (CAT), and superoxide dismutase 1 (SOD1) in the liver by activating the nuclear factor E2-related factor 2 (Nrf2) pathway. Oppositely, quercetin exhibited a powerful suppressive effect against key enzymes with oxidative activities, such as acetylcholinesterase (AChE) and butyrylcholinesterase (BChE) [35]. Thus, quercetin functions to strengthen the antioxidant defense system.

Quercetin has strong antioxidant and free radical scavenging capacities by removing ROS and inhibiting lipid peroxidation [36]. It is known that iron is a life-supporting micronutrient that is dispensable in the human diet and plays a crucial role in various fundamental metabolic processes. However, excessive iron accumulation can cause cell and tissue damage, since highly reactive iron catalyzes ROS generation. Quercetin effectively reduces iron deposition to inhibit lipid peroxidation and eliminates iron-catalyzed ROS production [37]. Based on the above evidence, quercetin exerts iron-chelating and ROS-scavenging activities.

Quercetin enhances the antioxidant defense system and reverses oxidative imbalance by modifying diverse signaling pathways. For instance, quercetin inhibited the activation of nuclear factor-*κ*B (NF-*κ*B) and mitogen-activated protein kinase (MAPK) signaling pathways and alleviated the oxidative stress induced by lipopolysaccharides/d-galactosamine (LPS/d-GalN) [38]. Quercetin stimulated Nrf2/glutamate-cysteine ligase (GCL)/GSH antioxidant signaling cascades [39]. As a result, quercetin increased the levels of cellular GSH and mitigated toosendanin-induced hepatotoxicity. Quercetin prevented lead-induced oxidative stress by limiting ROS generation [40]. Quercetin-mediated protective action might involve the activation of the phosphoinositide-3-kinase (PI3K)/phosphorylated protein kinase B (Akt) signal transduction pathway and the inhibition of the inositol-requiring enzyme 1 (IRE1)/c-Jun N-terminal kinase (JNK) signaling pathway.

It has been well documented that quercetin exhibits powerful anti-inflammatory effects and attenuates the inflammation process. The immunosuppressive capacities of quercetin are mediated by its effects on the levels of inflammatory cytokines, the generation of inflammation-producing enzymes (cyclooxygenase (COX) and lipoxygenase (LOX)), the maintenance of the stability of mast cells, and functional properties of immune cells (e.g., peripheral blood mononuclear cells and T cells) [2]. Quercetin shows broad-spectrum antimicrobial properties. It was reported that quercetin had a significant inhibitory effect on the growth of pathogenic microbes such as *Aspergillus flavus*, *Candida albicans*, *Escherichia coli*, *Listeria*, *Proteus*, *Pseudomonas aeruginosa*, *Salmonella enteritidis*, *Shigella*, and *Staphylococcus aureus* [41–43]. The antimicrobial mechanisms of quercetin involve the destruction of bacterial cell wall/membrane, alternation of cell permeability, regulation of protein synthesis and abundance, inhibition of enzymatic activities, and nucleic acid biosynthesis.

## 5. Noncoding RNAs

With the sequencing of the human genome, it became obvious that protein-coding genes only made up a minor part (~2%) of the human genome [44]. It has since then been a major challenge to decipher the remaining proportion of the genome. The advent of next-generation sequencing techniques greatly accelerates the characterization of the human transcriptome, revealing that a considerable fraction of the genome can be transcribed into ncRNAs. ncRNAs constitute a group of functional RNA molecules that usually do not encode proteins and instead directly function in the RNA form [45]. So far, many novel classes of ncRNAs have been discovered and characterized, which include miRNAs, siRNAs, small nucleolar RNAs (snoRNAs), piRNAs, lncRNAs, and circRNAs. It is widely accepted that ncRNAs function as vital modulators controlling gene expression at both transcriptional and posttranscriptional levels [46]. ncRNAs are also associated with gene production and genome reorganization [47]. Importantly, through their interaction with biomolecules (e.g., DNAs, RNAs, and proteins), ncRNAs are implicated in numerous physiological and pathological processes, including cell proliferation, differentiation, immune reactions, intercellular communications, infection, and carcinogenesis. Further investigation of ncRNAs, their target genes and potential linkage with various pathological processes would enrich our knowledge of the initiation and progression of important human diseases.

## 6. Roles of Main Noncoding RNAs in Cancer

According to their length, ncRNAs can be roughly classified into small ncRNAs (<200 nt) and lncRNAs (>200 nt) [11]. The small ncRNA species include miRNAs, siRNAs, and piRNAs, among which miRNAs have been prominently investigated for its important role in human development and disorders. By contrast, lncRNAs are typically greater than 200 nt in length and exert physiological functions by regulating gene expression at the transcriptional, posttranscriptional, and epigenetic levels. circRNAs are a special category of novel endogenous lncRNAs that are characterized by covalently closed single-stranded loop structures without free 5′ and 3′ ends. A large number of published works highlight the contribution of lncRNAs and circRNAs to cancer pathogenesis. The development of cancer is a complicated, multistep process, and ncRNAs involved in carcinogenesis may act as oncogenes or tumor suppressors. In recent years, ncRNAs have gained widespread attention for their abundance, expression patterns, functional roles in cancer, and potential clinical values.

### 6.1. MicroRNAs

**Figure 1:**
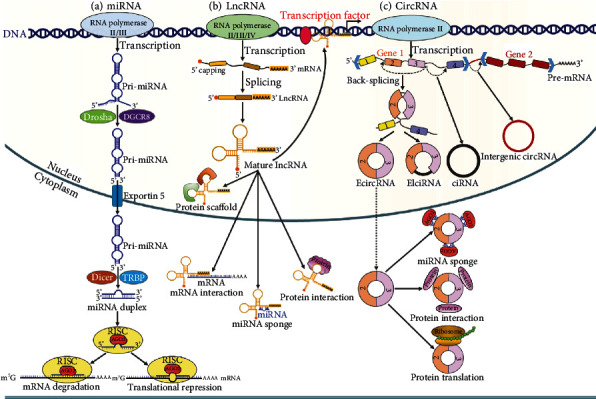
The biogenesis and main functions of miRNA, lncRNA, and circRNA. (a) Gene encoding miRNAs is transcribed by RNA polymerase (Pol) II/III, leading to the generation of primary miRNA (pri-miRNA). The pri-miRNA is cleaved by Drosha and DiGeorge syndrome critical region 8 (DGCR8). By cutting the stem of the pri-miRNA, a precursor miRNA (pre-miRNA) is produced. The pre-miRNA is then translocated to the cytoplasm via exportin 5. In the cytoplasm, the pre-miRNA is further processed by Dicer and transactivation response element RNA-binding protein (TRBP), which cleave the loop, the product being called miRNA duplex. One strand of the miRNA duplex, also referred to as the guide strand, is loaded onto an argonaute (AGO) protein to form the RNA-induced silencing complex (RISC). The miRNA directs RISC to the 3′ untranslated region (UTR) of target mRNAs by base pairing and participates in posttranscriptional gene regulation. (b) lncRNAs are transcribed by Pol II/III/IV from genomic loci with similar chromatin states to mRNAs. lncRNAs commonly have a 5′ cap and a 3′ poly(A) tail. The majority of lncRNAs are inefficiently processed and are retained in the nucleus, while only a small proportion of them undergo splicing and are exported to the cytoplasm. In the nucleus, lncRNAs regulate gene transcription by interacting with transcription factors. lncRNAs can also serve as the scaffold for proteins, thus modulating histone modification of various genes. Moreover, some lncRNAs function in the cytoplasm where they sequester miRNAs and interact with mRNAs or cytoplasmic proteins. (c) circRNAs are produced from the pre-mRNA through noncanonical splicing events called back-splicing. According to their origins, circRNAs can be divided into four main subtypes: exonic circRNAs (ecircRNAs), exon-intron circRNAs (EIciRNAs), intronic circRNAs (ciRNAs), and intergenic circRNAs. circRNAs have multiple functions, including sponging miRNAs, binding proteins, and encoding peptides or proteins.

miRNAs are by far the best-known class of ncRNAs in cancer. miRNAs belong to a group of endogenous, small ncRNAs of 17-25 nt in length. Gene encoding miRNAs is transcribed by RNA polymerase (Pol) II/III and processed via an evolutionarily conserved pathway [48] (Figure 1(a)). Specifically, a long primary transcript (pri-miRNA) shapes a characteristic hairpin structure that can be cleaved into a precursor miRNA (pre-miRNA) of approximately 60 nt in length by the microprocessor complex consisting of Drosha and DiGeorge syndrome critical region 8 (DGCR8) [49]. The produced pre-miRNA is then translocated to the cytoplasm via exportin 5 and Ran-GTP complex. The pre-miRNA is further processed by Dicer to form a miRNA duplex. One strand of the miRNA duplex, also referred to as the guide strand, is loaded onto an argonaute (AGO) protein to form the RNA-induced silencing complex (RISC). The miRNA directs RISC to the 3′ untranslated region (UTR) of target mRNAs by base pairing. miRNA-mediated gene silencing occurs through perfect or imperfect complementarity between miRNA recognition elements (MRE) and the 3′ UTR of their target mRNAs [50]. An individual miRNA can bind various mRNAs and, in turn, multiple miRNAs are able to jointly target the same mRNA [51]. Thus, it is not surprising that miRNAs can control many genes and signal transduction pathways.

Accumulating evidence has indicated the aberrant expression of numerous miRNAs in various cancers, implying that they may take part in cancer onset and development [52]. For instance, the expression level of miR-19a was remarkably higher in osteosarcoma tissues than that in noncancerous bone tissues [53]. Remarkably, high levels of miR-19a were associated with large tumor size, advanced clinical stage, tumor metastasis, and poor response to chemotherapy. miR-19a might be a novel prognostic marker for osteosarcoma patients. miR-339-5p was lowly expressed in gastric cancer (GC) tissues compared with adjacent normal tissues [54]. Decreased levels of miR-339-5p were closely correlated with cancer metastasis and poor prognosis in GC patients. The expression levels of serum miRNA let-7 were reduced in patients with breast cancer compared with the control group [55]. The levels of miRNA let-7 were negatively linked with tumor metastasis in patients with breast cancer. Mechanistic investigations indicate that miRNAs act as master regulators that govern the expression of potential oncogenes or tumor suppressor genes, thus affecting the diverse processes of cancer development. For instance, miR-155 drove tumorigenesis in different cancers by targeting tumor suppressors tumor protein 53 (TP53)-induced nuclear protein 1 (TP53INP1) and SH2 domain-containing inositol-5'-phosphatase 1 (SHIP1) [56]. miR-221 played an oncogenic function in cutaneous squamous cell carcinoma (CSCC) by targeting phosphatase and tensin homolog (PTEN) [57]. miR-214 was significantly downregulated in several types of cancer, including breast cancer, cervical cancer, GC, and nasopharyngeal carcinoma [58]. miR-214 suppressed tumor development by targeting the oncogene Slit-Robo Rho GTPase-activating protein 1 (*SRGAP1*). The expression level of miR-217 was decreased in pancreatic ductal adenocarcinoma (PDAC) tissues when compared with the corresponding normal pancreatic tissues [59]. Moreover, miR-217 modulated the expression of the oncogene *KRAS* and suppressed the growth of PDAC cells.

Hyperactivation of the Wnt/*β*-catenin signaling cascade is involved in occurrence and progression of human malignancies. Oncogenic miRNAs, such as miR-1229, miR-125b, miR-29a, and miR-3646, could promote tumor development and dissemination by triggering the Wnt/*β*-catenin signaling pathway [60]. On the contrary, several miRNAs (e.g., miR-135, miR-140-5p, miR-30a-5p, and miR-384) showed anticarcinogenic activity by blocking the Wnt/*β*-catenin pathway. The PI3K/Akt signaling pathway plays a role in modulating cancer cell proliferation, migration, and invasion [61]. The effects of miRNAs on cancer progression can be attributed to the deregulation of the PI3K/Akt signaling pathway. miR-21 and miR-425-5p facilitated cancer chemoresistance and growth by inhibiting the PI3K/Akt signaling, while miR-93, miR-106b, and miR-301 promoted cancer cell proliferation and invasion by activating the PI3K/Akt signaling [62]. The Notch signaling pathway elicits oncogenic or tumor-suppressive roles in human malignancies [63]. miR-223 and miR-1275 promoted cancer occurrence and development by activating the Notch signaling pathway [64, 65]. miR-1271-5p and miR-34 suppressed the proliferation, invasion, and migration of cancer cells by blocking the Notch signaling cascade [66, 67]. miR-139-5p and miR-199b-5p could sensitize cancer cells to chemotherapeutic agents by reducing the activity of the Notch1 signaling [68, 69].

Collectively, the biological functions of aberrantly expressed miRNAs in cancer pathogenesis such as cell proliferation, invasion, migration, and chemoresistance have been systematically explored. The coordination of cancer-relevant genes and signaling pathways by miRNAs constitutes the main cause of their implication in cancer development. However, there is still a long way to go before a thorough understanding of the connection between miRNAs and cancer biology. A multitude of studies have moved toward the exploitation of miRNAs in cancer treatment as a novel strategy to disrupt oncogenic processes. New in silico approaches for the design and generation of artificial miRNAs as well as new platforms for miRNA delivery have been developed in the past years, which represent a research frontier in miRNA-based cancer therapies.

### 6.2. Long Noncoding RNAs

lncRNAs are ubiquitously defined as RNA molecules greater than 200 nt in length with no or low protein-coding capacity and form the vast majority of the ncRNAs. Based on their locations and characteristics, lncRNAs can be divided into five types: intronic, sense, antisense, bidirectional, and intergenic [70]. lncRNAs are mainly transcribed by Pol II from genomic loci with similar chromatin states to mRNAs [71]. lncRNAs commonly have a 5′ cap and a 3′ poly(A) tail (Figure 1(b)). Intriguingly, active enhancer and promoter regions can also be transcribed into lncRNAs, known as enhancer RNAs (eRNAs) and promoter upstream transcripts (PROMPTs), respectively [72, 73]. Compared with mRNAs, the majority of lncRNAs are inefficiently processed and are retained in the nucleus, while only a small proportion of them undergo splicing and are exported to the cytoplasm [74]. lncRNAs are featured by paucity of introns and low GC content, which result in their low abundance within the cell [75]. It is worthwhile noting that lncRNA genes are less evolutionarily conserved and contain fewer and longer exons. lncRNAs exhibit tissue- and cell type-specific expression patterns and play an important role in various cellular events, such as splicing, transcription, translation, and epigenetic regulation [76]. Functionally, lncRNAs can be classified as decoy, guide, scaffold, and signaling lncRNAs [77]. Decoy lncRNAs act as molecular sinks for transcription factors and repressors, RNA molecules, and RNA-binding proteins (RBPs). Decoy lncRNAs regulate transcription and translation by titrating these effector factors away from binding to their intrinsic targets. Guide lncRNAs target the regulatory or enzymatically active protein complexes and direct them to particular target gene promoters or genomic location modulating downstream signaling events and gene expression [78]. Scaffold lncRNAs work as a central platform for the assembly of diverse protein complexes and can be directed to target gene promoter or particular genomic location, thereby affecting gene expression and chromosomal dynamics. Signaling lncRNAs are linked with several signaling pathways and their abundance reflects a dynamic signaling episode. Additionally, lncRNAs are able to orchestrate the pivotal functions of other ncRNAs such as miRNAs and snoRNAs.

lncRNAs have emerged as versatile players in gene regulation in multiple biological and physiopathological contexts, particularly cancer [79]. The number of lncRNAs involved in carcinogenesis and cancer progression is exponentially increasing. lncRNAs may show tumor-promoting and -suppressive functions. The HOX transcript antisense intergenic RNA (HOTAIR), one of the most well-characterized lncRNAs, was highly expressed in various types of cancer [80]. HOTAIR facilitated tumorigenesis and tumor development by regulating multiple molecules (e.g., vascular endothelial growth factor (VEGF) and E-cadherin and matrix metalloproteinase-9 (MMP-9)) that were connected with epithelial-to-mesenchymal transition (EMT), tumor invasion, and metastasis. Mechanistically, HOTAIR was involved in tumor progression by sponging diverse miRNAs such as miR-143-3p, miR-148a, miR-20a-5p, miR-206, miR-23b, and miR-214/miR-217. HOTAIR also regulated cancer-related pathways, including the p53/p21 signaling, the PI3K/Akt/mTOR signaling cascade, the Notch/Wnt pathway, and the Wnt/*β*-catenin signaling pathway. Therefore, HOTAIR played a promotive role in cancer cell proliferation, invasion, metastasis, and chemoresistance. H19 was upregulated in cancer patients compared with controls [81]. A growing body of evidence has validated the oncogenic effects of H19. H19 controlled the key processes in malignant transformation and cancer progression, including cancer cell proliferation, migration, invasion, and EMT process. H19 acted as a competitive endogenous RNA (ceRNA) for different miRNAs (e.g., let-7a, miR-152, miR-194-5p, and miR-675). The regulation of signaling pathways, such as the JNK pathway, the Wnt signaling, and apoptotic signaling pathway, also contributed to the protumorigenic effects of H19. Metastasis-associated lung adenocarcinoma transcript 1 (MALAT1), one of the most abundant lncRNAs in normal tissues, was abnormally expressed in human malignancies [82]. MALAT1 can act multifaceted roles of oncogenes and tumor suppressors in cancer. MALAT1 functioned through decoying various miRNAs, including miR-1, miR-145, miR-200c, miR-202, miR-204, and miR-206 [82]. As a result, MALAT1 altered cancer cell proliferation, migration, invasion, and sensitivity to therapeutics. Moreover, MALAT1 caused the silencing of genes intertwined with cancer progression and metastasis by recruiting chromatin-modifying complexes to target gene loci (e.g., *E-cadherin* and *PCDH10*) [83, 84]. MALAT1 was able to control cancer cell EMT, migration, invasion, and metastasis by orchestrating the Wnt signaling [85]. In some cancers, the MALAT1*-*PI3K/Akt axis was involved in cancer metastasis, and the NF-*κ*B pathway mediated the effect of MALAT1 on the EMT process and chemoresistance in cancer cells [86, 87]. Additionally, MALAT1 affected cancer cell proliferation through its regulation of the MAPK signaling [88]. Maternally expressed gene 3 (MEG3) was a tumor suppressor lncRNA which activated the p53 signaling [89]. MEG3 governed several cancer-relevant signal transduction pathways, such as TGF-*β*, PI3K/Akt, Notch, mTOR, and Wnt/*β*-catenin signaling cascades. MEG3 could enhance the sensitivity of cancer cells to chemotherapeutic agents by downregulating oncogenic miR-21-5p and upregulating its downstream target sex-determining region Y-box 7 (SOX7) [90].

### 6.3. Circular RNAs

circRNAs are a special class of naturally occurring endogenous lncRNAs with widespread distribution and multiple functions [91]. circRNAs are produced from the pre-mRNA through noncanonical splicing events called back-splicing, whereby the upstream 3′ splice acceptor is covalently connected with the downstream 5′ splice donor [92] (Figure 1(c)). circRNAs form a unique covalently closed loop without 5′ end cap structures and 3′ end poly(A) tails. In comparison with linear RNAs, circRNAs display high stability due to their circular configuration. They can be derived from exons in the coding region of a gene, the 5′ or 3′ UTRs, introns, intergenic regions, and antisense RNAs [93]. Thus, circRNAs are divided into four main subtypes: exonic circRNAs (ecircRNAs), exon-intron circRNAs (EIciRNAs), intronic circRNAs (ciRNAs), and intergenic circRNAs. The biogenetic mechanisms of distinct subclasses of circRNAs are diversely coordinated. Formation of ecircRNAs may be achieved by lariat-driven circularization, RBP-mediated circularization, and intron-pairing-driven circularization [94]. In the lariat-driven circularization model, RNA folding occurs during pre-mRNA transcription, and exons are skipped along with folding of the RNA [95]. The structural alternations cause the generation of lariats, in which initially discontinuous exons become very close to each other as the interior changes. The intronic sequences in the lariat are spliced out, and the exons are joined by a 5′-3′ phosphodiester bond to form ecircRNAs. In the RBP-mediated circularization model, RBPs bridge the two flanking introns and connect them together, resulting in the formation of ecircRNAs [96]. During intron-pairing-driven circularization, the base paring of reverse complementary sequences within introns on both sides of the pre-mRNA mediates the generation of circRNAs [97]. In some cases, the introns of pre-mRNAs can be retained to form EIciRNAs. ciRNAs are produced from lariat introns that escape from the debranching step of canonical linear splicing. The formation of ciRNAs primarily relies on a consensus motif of a 7 nt GU-rich element adjacent to 5′ splice site and an 11 nt C-rich element near the branchpoint site [98].

circRNAs exert a variety of biological functions, which include functioning as miRNA sponges, sequestering RBPs, regulating alternative splicing/transcription and gene expression, and encoding functional proteins/peptides [99]. These characteristics suggest that circRNAs serve a key role in biological cellular events and pathological processes. The bulk of evidence proves that circRNAs have a close relationship with the pathology of many diseases, including cancer, diabetes, and cardiovascular diseases [100]. Particularly, a number of cancer-associated circRNAs have been identified and characterized. circRNAs act as important players in cancer onset and progression by sequestering miRNAs, interacting with proteins or producing functional proteins. Reportedly, hsa_circ_0025202 showed low expression in breast cancer tissues compared with noncancerous tissues [101]. hsa_circ_0025202 acted as a miRNA sponge for miR-182-5p and governed the expression of forkhead box protein O3 (Foxo3). Accordingly, hsa_circ_0025202 repressed cancer cell proliferation and migration and enhanced the chemotherapeutic sensitivity of cancer cells. CircFoxo3 induced cell cycle arrest and suppressed cancer cell proliferation by combining with cell cycle-related proteins, cyclin-dependent kinase 2 (CDK2), and cyclin-dependent kinase inhibitor 1 (p21) [102]. Circ-SHPRH was lowly expressed in glioblastoma and could encode for a 17 kDa protein termed SHPRH-146aa [103]. SHPRH-146aa protected its linear counterpart from ubiquitin-mediated degradation, resulting in the suppression of cancer cell proliferation and tumorigenicity. Collectively, aberrant expression of circRNAs has an impact on the biological behaviors of cancer cells, such as cell proliferation, apoptosis, and metastasis. Additional investigations are warranted to discover more cancer-related circRNAs and to disclose the functional roles of circRNAs in cancer.

## 7. Targeting ncRNAs by Quercetin in Different Types of Cancers

Dietary phytochemicals with anticancer properties have been gaining focus for cancer treatment since they show highly effective in preventing cancer. Quercetin exerts anticarcinogenic effects via diverse mechanisms. Quercetin can inhibit cancer cell proliferation, induce cell apoptosis, drive cell cycle arrest, and repress mitotic processes by regulating cyclins, proapoptotic/antiapoptotic proteins, and diverse signaling cascades [104]. Quercetin may represent a promising agent for the prevention and treatment of cancer. The association between ncRNAs and cancer biology has been gradually elucidated, underlining that ncRNAs may be an effective target for cancer intervention. Remarkably, quercetin exhibits the activity to regulate the levels of cancer-relevant ncRNAs, thus suppressing carcinogenesis and cancer progression (Table 1).

### 7.1. Blockade of Carcinogenesis by Quercetin

**Figure 2:**
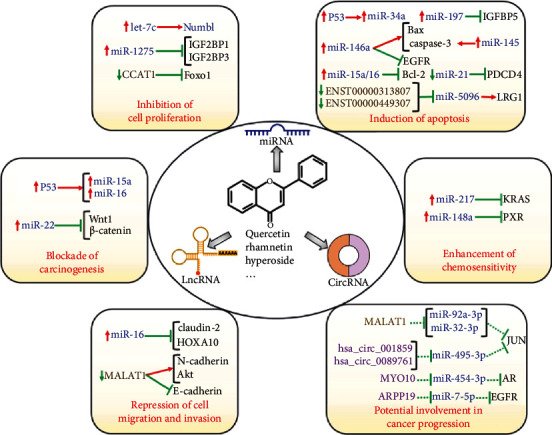
Anticancer effects of quercetin and its derivatives by regulation of ncRNAs. ncRNAs participate in key processes during cancer development and progression by modulating their target genes or affecting the activity of cancer-related signaling cascades. Thus, quercetin plays an important role in controlling carcinogenesis, cancer cell proliferation, apoptosis, chemosensitivity, migration, and invasion via alteration of the expression and function of cancer-relevant ncRNAs.

Quercetin regulates carcinogenesis by interacting with tumor suppressor miRNAs (Figure 2). Methoxylated quercetin glycoside inhibited the tumorigenesis and proliferation of hepatocellular carcinoma (HCC) cells [105]. Methoxylated quercetin glycoside increased the expression levels of TP53 (p53) and its downstream tumor-suppressive miRNAs that included miR-15a and miR-16. Thus, methoxylated quercetin glycoside exerted an anticarcinogenic role in HCC by inducing the TP53/miR-15a/miR-16 axis. Likewise, another study showed that quercetin exerted proapoptotic effects in HCC cells [106]. Quercetin could enhance miR-34a expression through upregulation of p53. miR-34a directly targeted sirtuin 1 (SIRT1), which was able to inactivate the p53 signaling pathway. The miR-34a/SIRT1 axis served as a feedback loop to increase the activity of p53 and thus promoted the p53-related apoptosis signal. Collectively, miR-34a played a critical role in the anticarcinogenic effects of quercetin in HCC. Quercetin controls the expression and activity of miRNAs by regulating their upstream molecules. Further study is required to investigate whether quercetin can directly target cancer-relevant miRNAs. It was reported that quercetin elevated the expression of miR-22 and lowered the expression of Wnt1 and *β*-catenin in oral squamous cell carcinoma (OSCC) cells [24]. The effect of quercetin on the Wnt1/*β*-catenin signaling was overturned by miR-22 inhibitor. Thus, silencing of miR-22 abated tumor-suppressive effects of quercetin in OSCC cells. Altogether, quercetin counteracted OSCC carcinogenesis by affecting the miR-22/Wnt1/*β*-catenin signaling cascade.

### 7.2. The Antiproliferative Effects of Quercetin in Cancer

Quercetin plays a negative role in cancer cell proliferation by targeting miRNAs (Figure 2). miRNA let-7c was downregulated in pancreatic cancer [107]. let-7c could activate Numbl through posttranscriptional upregulation of its mRNA levels and downregulation of Notch. let-7c repressed the colony formation of pancreatic cancer cells. Quercetin inhibited pancreatic cancer growth by upregulating let-7c and activating Numbl. The protumorigenic insulin-like growth factor-2 binding proteins (IGF2BPs) were highly expressed in HCC and could be directly targeted by the tumor suppressor miR-1275 [108]. Quercetin was reported to reduce the viability of HCC cells by upregulating miR-1275 and reducing the expression levels of IGF2BP1 and IGF2BP3.

### 7.3. Induction of Cellular Apoptotic Pathways by Quercetin

Quercetin modulates the abundance of miRNAs that target proapoptotic or antiapoptotic proteins. Quercetin inhibited the viability and proliferation and promoted the apoptosis in meningioma cells [23]. In terms of mechanism, quercetin downregulated antiapoptotic B-cell lymphoma-2 (Bcl-2) and upregulated proapoptotic Bcl-2-associated X protein (Bax). The antiapoptotic insulin-like growth factor-binding protein 5 (IGFBP5) was a direct target of miR-197. Consistently, quercetin enhanced the expression of miR-197 and reduced the expression of IGFBP5. These findings indicated that the impact of quercetin on both antiapoptotic and proapoptotic factors represented a possible mechanism associated with its anticancer property. It should be noted that extensive studies and clinical trials should be carried out to determine the therapeutic benefits of quercetin for cancer treatment. Likewise, quercetin exhibited growth-inhibitory effects on breast cancer cells [109]. miR-146a was found to be remarkably upregulated after quercetin treatment. miR-146a overexpression magnified the antiproliferative activity of quercetin. Oppositely, loss of miR-146a reversed the inhibitory effects of quercetin on breast cancer cell proliferation. Mechanistically, miR-146a upregulation resulted in elevated expression of Bax and cleaved caspase-3 and decreased expression of epidermal growth factor receptor (EGFR). Thus, quercetin induced the apoptosis and inhibited the invasion and metastasis of breast cancer cells by raising miR-146a levels. The exact mechanism of how quercetin regulated miR-146a expression needs further clarification. It was reported that quercetin promoted apoptosis of radio-resistant B-1 cells by elevating miR-15a/16 expression and reducing Bcl-2 expression [110]. Radio-resistant B-1 cells showed similarities to B-chronic lymphocytic leukemia (B-CLL) cells. Quercetin might be used as a promising chemotherapeutic agent for B-CLL treatment. However, it remains to verify whether quercetin exerted a proapoptotic effect on B-CLL cells. In addition, quercetin inhibited the growth of ovarian cancer cells [111]. The expression level of miR-145 was reduced in ovarian cancer cells. Quercetin induced the expression of miR-145 and thus caused the activation of caspase-3. miR-145 depletion weakened the inhibitory effect of quercetin on ovarian cancer growth. Therefore, miR-145 might be crucial in quercetin-induced apoptosis in ovarian cancer cells.

Quercetin and hyperoside in combination (QH) facilitated the apoptosis of prostate cancer cells by stimulating the expression of caspase-3 and the cleavage of poly(adenosine diphosphate ribose) polymerase (PARP) [112]. QH also exerted anti-invasive and antimigratory effects on prostate cancer cells. It turned out that QH decreased miR-21 expression, thus enhancing the expression of the tumor suppressor programmed cell death protein 4 (PDCD4). On the contrary, upregulation of miR-21 could significantly diminish the protective role of QH in prostate cancer. Quercetin inhibited the viability and induced the apoptosis of colon cancer cells [113]. This effect was partially explained by quercetin-induced dysregulation of ncRNAs (miRNAs, lncRNAs, and circRNAs). Among the altered miRNAs, miR-125b-2-3p, miR-320b, miR-320c, miR-320d, and miR-338-3p were markedly upregulated in colon cancer cells treated with quercetin. These miRNAs might play a pivotal role in the anticancer mechanism of quercetin. Further experimental studies are required to elucidate the roles and molecular mechanisms of the dysregulated miRNAs in quercetin-mediated inhibition of cancer development.

### 7.4. Inhibitory Effects of Quercetin on Cancer Cell Migration and Invasion

Claudin-2 was upregulated in human lung adenocarcinoma tissues [114]. Downregulation of claudin-2 suppressed the proliferation and migration of lung adenocarcinoma cells. Quercetin reduced claudin-2 expression by elevating miR-16 expression. The chemotherapeutic potential of quercetin might be partially ascribable to its regulatory effects on claudin-2 expression. Quercetin was found to reduce the viability, migration, and invasion of oral cancer cells [115]. miR-16 was downregulated in oral cancer tissues compared with peritumor tissues. Quercetin could increase the level of miR-16 in oral cancer cells. miR-16 exerted an anticarcinogenic function by targeting homeobox A10 (HOXA10). Importantly, miR-16 depletion abrogated the inhibitory effects of quercetin on cancer development. Thus, quercetin prevented cell viability, migration, and invasion in oral cancer cells by enhancing miR-16 expression and attenuating HOXA10 level.

### 7.5. Contribution of Quercetin to Enhanced Chemosensitivity of Cancer Cells

Quercetin was capable of sensitizing osteosarcoma cells to cisplatin [116]. The expression level of miR-217 was increased in osteosarcoma cells after quercetin and/or cisplatin exposure, whereas that of its downstream target KRAS was decreased. Silencing of miR-217 reduced cisplatin sensitivity of quercetin-treated osteosarcoma cells. By contrast, upregulation of miR-217 led to the opposite effects. These results suggested that the miR-217/KRAS axis was involved in quercetin-regulated cisplatin sensitivity in osteosarcoma. Rhamnetin, a methylated derivative of quercetin, was found to inhibit Sirt1 activation by combining with its hydrophobic pocket, thus promoting acetylation at lysine residue 373 of p53 [117]. Acetylation of p53 was essential for the activation of its transcription factors and enhanced miRNA transcription. Rhamnetin could also facilitate the recruitment of p53 to the promoter region of miR-148a. As a result, rhamnetin induced the expression of miR-148a. This event caused the downregulation of pregnane X receptor (PXR) and its drug resistance-associated downstream genes (cytochrome P450 3A4 (*cyp3a4*) and multidrug resistance gene 1 (*mdr-1*)), which mediated the metabolism or expulsion of sorafenib in HCC cells. Collectively, rhamnetin blocked the metabolic clearance of sorafenib in HCC cells and increased the sensitivity of HCC cells to sorafenib. Rhamnetin could serve as a potential adjuvant agent for overcoming sorafenib resistance in HCC therapy.

### 7.6. Anticancer Actions of Quercetin Mediated by lncRNAs and circRNAs

Phytochemicals, including curcumin, quercetin, and resveratrol, have emerged as important regulators of lncRNAs in different types of cancers [6]. Hyperoside is a flavonol glycoside compound and exhibits anticancer activities against various cancers [118]. Foxo1 played a key role in cancer cell apoptosis. The expression level of Foxo1 was decreased, while that of lncRNA colon cancer associated transcript 1 (CCAT1) was elevated in non-small-cell lung cancer (NSCLC) cells. Hyperoside was able to enhance Foxo1 expression by downregulating CCAT1 in NSCLC cells. Further investigation showed that hyperoside repressed the proliferation and promoted the apoptosis of NSCLC cells via the CCAT1-mediated Foxo1 signaling. The expression level of MALAT1 was remarkably diminished in quercetin-treated prostate cancer cells [21]. MALAT1 played an oncogenic role in cancer progression. Mechanistically, MALAT1 promoted the EMT process in prostate cancer cells by upregulating N-cadherin and downregulating E-cadherin. Moreover, MALAT1 activated the PI3K/Akt signaling cascade by increasing the expression level of phosphorylated Akt. Quercetin inhibited cell proliferation, invasion, and migration but facilitated apoptosis in prostate cancer cells via blocking the EMT process and the PI3K/Akt signaling cascade by targeting MALAT1. Oppositely, MALAT1 overexpression conferred chemoresistance to prostate cancer cells against the cytotoxicity of quercetin. Thus, MALAT1 could antagonize the tumor-suppressive effects of quercetin. These results suggested that MALAT1 could be a pivotal target in quercetin treatment of prostate cancer, providing a novel molecular basis for the clinical use of quercetin in treating prostate cancer.

Quercetin inhibited the proliferation and induced the apoptosis of colon cancer cells by regulating the expression of several lncRNAs (Figure 2) [113]. Quercetin had the ability to downregulate leucine-rich *α*-2-glycoprotein-1 (LRG1), which might explain its role as an anticancer compound. The lncRNAs ENST00000313807 and ENST00000449307 were able to increase LRG1 expression through competitive interaction with miR-5096. It was likely that quercetin induced colon cancer cell apoptosis by inhibiting LRG1 expression via downregulating ENST00000313807 and ENST00000449307. In addition, the circRNA (8: 93786223|93822563) was predicted to regulate LRG1 expression by competitively combining with miR-5096 [113]. The role and mechanisms of action of the circRNA/miRNA/LRG1 axis in quercetin-mediated tumor inhibition deserve further investigation.

Quercetin suppressed the proliferation of cervical cancer cells [119]. The combined analyses of the Gene Expression Omnibus (GEO) database and RNA-sequencing results indicated that quercetin altered the expression of several genes, including Jun protooncogene (*JUN*), androgen receptor (*AR*), and *EGFR*. Further exploration of the upstream interacting ncRNAs of these genes identified one lncRNA (MALAT1) and seventy-one circRNAs (e.g., hsa_circ_001859, hsa_circ_0089761, MYO10, and ARPP19). The lncRNA/circRNA-miRNA-mRNA regulatory networks might take part in the antagonistic effects of quercetin against cervical cancer. These results might help provide valuable diagnostic biomarkers and therapeutic targets for cervical cancer. However, it is of great necessity to determine the genuine roles and clinical significance of the identified ncRNAs in cervical cancer.

## 8. Conclusions and Future Perspectives

Quercetin, a natural compound occurring in diet, has been reported to possess antioxidant, antimicrobial, and anti-inflammatory effects. Because of its multifaceted pharmacological activities, quercetin has grabbed a great deal of attention. Importantly, a growing body of evidence has proven the cancer preventive role of quercetin. Quercetin shows a broad spectrum of anticancer properties such as proapoptotic, antiproliferative, anti-invasive, and antimigratory effects. Recent studies have shown that quercetin can regulate the expression and activity of ncRNAs, including miRNAs, lncRNAs, and circRNAs. With important roles in numerous cellular processes, it is not surprising that the deregulation of ncRNAs has been closely linked to cancer development. Thus, quercetin-mediated modulation of ncRNAs is a contributory factor to its anticarcinogenic potential, which revolutionizes our perception of the mechanisms of action of quercetin. Since the ncRNA field is a new frontier of biomedical research with limited clinical data, advanced integrative studies are critically needed to delineate the chemotherapeutic potential of quercetin by virtue of ncRNA regulation and mechanisms.

With accumulating evidence on the anticancer efficacy of quercetin, there is a clear need to fill the knowledge gaps in translating basic research findings to clinical practice. To achieve this goal, several key challenges for future research directions need to be addressed. First, molecular mechanisms responsible for the regulation of ncRNAs have been characterized for diverse bioactive polyphenols, including direct interaction, transcriptional and epigenetic modulation, and fine-tuning of ncRNA biogenesis. Nevertheless, there are few data available on quercetin. Thus, it remains to characterize how quercetin influences the function of ncRNAs. Second, quercetin can regulate various molecules and signaling pathways through its effects on the upstream ncRNAs. Quercetin is found to target different types of ncRNAs. circRNAs or lncRNAs modulate gene expression by competitively combining with miRNAs, constituting complex ceRNA regulatory circuits. It is possible that quercetin exerts anticancer effects by manipulating intricate ceRNA regulatory networks. At present, there is limited information about the impact of quercetin on lncRNA and circRNA expression profiles in cancer. It is necessary to comprehensively characterize the landscape of lncRNAs and circRNAs associated with the anticancer activity of quercetin. The in-depth investigation on quercetin-related ncRNA transcriptomic signatures in cancer will facilitate the elucidation of molecular mechanisms underlying the tumor-suppressive action of quercetin. Third, considering that quercetin metabolites may be potential bioactive substances, more efforts should be made to uncover the biological functions of quercetin derivatives. Further study on quercetin and its metabolites will expedite the discovery and development of improved chemotherapeutic agents for cancer treatment. Finally, albeit the potential of quercetin to treat cancer, its poor bioavailability and the requirement for high doses have a negative impact on its clinical efficacy. As nano-based formulations can enhance the bioavailability and particular targeting of natural compounds, their use may attenuate the dosage and improve the therapeutic effectiveness of quercetin [120]. Collectively, bioactive quercetin is widely available and has various health benefits with limited side effects. Quercetin holds great potential to become an effective agent for cancer prevention and treatment or an adjuvant agent in combination with conventional anticancer therapies. Quercetin is capable of targeting various ncRNAs that need to be further deciphered to adequately understand how this bioactive compound could be useful in cancer intervention.

## Figures and Tables

**Table 1:** Modulation of different ncRNAs by quercetin and its derivatives in cancer.

Biological process	Quercetin type	Target ncRNA	Cancer type	Expression	Mechanism	Reference
Anticarcinogenesis	Methoxylated quercetin glycoside	miR-15a, miR-16	Hepatocellular carcinoma	↑	Suppress the expression of oncogenes (e.g., Bcl-2, CCND1, and Mcl-1)	[105]
Proapoptosis	Quercetin	miR-34a	Hepatocellular carcinoma	↑	Inactivate the p53 signaling pathway by targeting SIRT1	[106]
Anticarcinogenesis	Quercetin	miR-22	Oral squamous cell carcinoma	↑	Impede the Wnt1/*β*-catenin signaling cascade	[24]
Antiproliferation	Quercetin	let-7c	Pancreatic cancer	↑	Upregulate Numbl and downregulate Notch	[107]
Antiproliferation	Quercetin	miR-1275	Hepatocellular carcinoma	↑	Suppress the expression of IGF2BP1 and IGF2BP3	[108]
Proapoptosis	Quercetin	miR-197	Meningioma	↑	Suppress the expression of IGFBP5	[23]
Proapoptosis, anti-invasion, and antimetastasis	Quercetin	miR-146a	Breast cancer	↑	Upregulate Bax and cleaved caspase-3; downregulate EGFR	[109]
Proapoptosis	Quercetin	miR-15a, miR-16	B-chronic lymphocytic leukemia	↑	Suppress the expression of Bcl-2	[110]
Proapoptosis	Quercetin	miR-145	Ovarian cancer	↑	Induce the activation of caspase-3	[111]
Antimigration, anti-invasion	Quercetin, hyperoside	miR-21	Prostate cancer	↓	Suppress the expression of PDCD4	[112]
Proapoptosis	Quercetin	miR-125b-2-3p, miR-320b, miR-320c, miR-320d, and miR-338-3p	Colon cancer	↑	Unknown	[113]
Antiproliferation, antimigration	Quercetin	miR-16	Lung adenocarcinoma	↑	Suppress the expression of claudin-2	[114]
Antimigration, anti-invasion	Quercetin	miR-16	Oral cancer	↑	Suppress the expression of HOXA10	[115]
Chemosensitization	Quercetin	miR-217	Osteosarcoma	↑	Suppress the expression of KRAS	[116]
Chemosensitization	Rhamnetin	miR-148a	Hepatocellular carcinoma	↑	Suppress the expression of PXR and drug resistance-associated genes (e.g., *cyp3a4* and *mdr-1*)	[117]
Antiproliferation, proapoptosis	Hyperoside	CCAT1	Non-small-cell lung cancer	↓	Suppress the expression of Foxo1	[118]
Antimigration, anti-invasion	Quercetin	MALAT1	Prostate cancer	↓	Upregulate N-cadherin and phosphorylated Akt; downregulate E-cadherin	[21]
Proapoptosis	Quercetin	ENST00000313807, ENST00000449307	Colon cancer	↓	Enhance the expression of LRG1 by targeting miR-5096	[113]
Antiproliferation	Quercetin	MALAT1, hsa_circ_001859, hsa_circ_0089761, MYO10, and ARPP19	Cervical cancer	—	Alter the expression of *JUN*, *AR*, and *EGFR*	[119]

## Data Availability

It is a review paper and no data is included.
